# Multi-platform whole genome sequencing for tuberculosis clinical and surveillance applications

**DOI:** 10.1038/s41598-024-55865-1

**Published:** 2024-03-03

**Authors:** Joseph Thorpe, Waritta Sawaengdee, Daniel Ward, Monica Campos, Nuanjun Wichukchinda, Boonchai Chaiyasirinroje, Aungkana Thanraka, Jaluporn Chumpol, Jody E. Phelan, Susana Campino, Surakameth Mahasirimongkol, Taane G. Clark

**Affiliations:** 1 Department of Infection Biology, Faculty of Infectious and Tropical Diseases, London School of Hygiene and Tropical Medicine, Keppel Street, London, WC1E 7HT UK; 2 Department of Medical Sciences, Medical Genetics Center, Medical Life Sciences Institute, Ministry of Public Health, Nonthaburi, 11000 Thailand; 3 TB/HIV Research Foundation (THRF), Chiang Rai, 57000 Thailand; 4 Department of Medical Technology, Chiangrai Prachanukroh Hospital, Chiang Rai, 57000 Thailand; 5 The Office of Disease Prevention and Control 7, Khon Kaen, 40000 Thailand; 6 Faculty of Epidemiology and Population Health, London School of Hygiene and Tropical Medicine, London, WC1E 7HT UK

**Keywords:** Genomics, Sequencing, Mycobacterium, *Pe/ppe* genes, Transmission, Tuberculosis, Infectious-disease diagnostics, Sequencing, Genome informatics, Bacterial genetics, Antibacterial drug resistance

## Abstract

Whole genome sequencing (WGS) of *Mycobacterium tuberculosis* offers valuable insights for tuberculosis (TB) control. High throughput platforms like Illumina and Oxford Nanopore Technology (ONT) are increasingly used globally, although ONT is known for higher error rates and is less established for genomic studies. Here we present a study comparing the sequencing outputs of both Illumina and ONT platforms, analysing DNA from 59 clinical isolates in highly endemic TB regions of Thailand. The resulting sequence data were used to profile the *M. tuberculosis* pairs for their lineage, drug resistance and presence in transmission chains, and were compared to publicly available WGS data from Thailand (n = 1456). Our results revealed isolates that are predominantly from lineages 1 and 2, with consistent drug resistance profiles, including six multidrug-resistant strains; however, analysis of ONT data showed longer phylogenetic branches, emphasising the technologies higher error rate. An analysis incorporating the larger dataset identified fifteen of our samples within six potential transmission clusters, including a significant clade of 41 multi-drug resistant isolates. ONT's extended sequences also revealed strain-specific structural variants in *pe/ppe* genes (*e.g. ppe50*), which are candidate loci for vaccine development. Despite some limitations, our results show that ONT sequencing is a promising approach for TB genomic research, supporting precision medicine and decision-making in areas with less developed infrastructure, which is crucial for tackling the disease’s significant regional burden.

## Introduction

Tuberculosis (TB), caused by bacteria in the *Mycobacterium tuberculosis complex*, is a global infectious disease, causing 10.6 million cases and 1.6 million associated deaths in 2021 alone, with nearly two-thirds of new cases in Asia^1^. Disease control in Asia is being compromised by undetected bacterial resistance to anti-TB drugs, making early diagnosis, appropriate therapy choice, and active case finding important measures to minimise the transmission of strains that limit treatment choices. However, these approaches have not been applied systematically across the globe. Genomic variants in *M. tuberculosis* drug targets or pro-drug activators, including single nucleotide polymorphisms (SNPs) and small insertions and deletions (indels), are responsible for drug resistance (DR). In developed countries, the lowering cost and implementation of next generation sequencing technologies (NGS) are revolutionising the diagnosis and clinical management of TB, through bacterial “profiling” using the genetic data, including of DR. High burden TB countries in Asia, including Thailand are seeking to adopt this approach as part of clinical care and public health management, and there is substantial investment in genomics infrastructure.

The *M. tuberculosis* complex is phylogeographically distributed in defined lineages that can determine the emergence of DR, transmissibility, pathogenicity, disease site and severity^2^. Drug resistant *M. tuberculosis* is one of the major threats to effectively control the disease, especially resistance to first-line rifampicin (RR-TB) and isoniazid (HR-TB); together, called multi-drug resistance (MDR-TB). In Thailand, the estimated proportion of MDR-TB cases among new cases is 1.7%, while among previously treated cases it is 9.8%. Globally, an estimated 3.6% of new TB cases and 18% of previously treated cases had MDR-TB in 2021^1^. Successful worldwide efforts to decrease TB burden have focused on advanced algorithms for early diagnosis, appropriate therapy choice and active case finding. Whilst diagnostics endorsed for TB and DR detection (e.g., Xpert MTB/RIF, XDR) are rapid compared to laboratory “phenotypic” drug susceptibility tests (DSTs), they are costly and do not capture all genetic mutations required for precise management of advanced forms of DR. Treatment programs involving DR in high incidence countries are keen to implement genomic tools, but the robust evaluation of clinical outcomes is needed to demonstrate efficacy and develop clear, simple guidelines for clinical application. Recent successes in developed countries have been led by advances in NGS (e.g., Illumina, Oxford Nanopore (ONT)), with increasing opportunities to use these directly from sputum or DNA from limited *M. tuberculosis* culture (MGIT), in near real time, at decreasing costs.

Whole genome sequencing (WGS) data generated by NGS Illumina platforms, can be used to profile *M. tuberculosis* for DR, and lineages using SNPs or indels^3^. Transmission events can be inferred through identification of variants in *M. tuberculosis* isolates sourced from different patients with (near) identical genomes. Characterising the phylogeographic distribution of *M. tuberculosis* strains across regions can reveal outbreaks of more virulent lineages, including Beijing strains. These WGS analyses have been made possible through advances in health informatics, including *M. tuberculosis* profiling tools (e.g., TB-Profiler^3^). Relatedly, ONT platforms are gaining traction for genomic investigations, and their portability make them implementable in resource poor settings. However, the platform has a known higher error rate, and although there is evidence that DR can be inferred^4^, its suitability for transmission analysis is less clear.

With NGS gaining traction, ONT and other rapid platforms are expected to play a key role in the fight against TB. Here, in a paired sequencing analysis of 59 *M. tuberculosis* replicate DNA sourced from Thailand, we compare WGS data from ONT and Illumina platforms, and evaluate concordance in variant calls and the positioning of isolates on a phylogenetic tree, which can provide insights into transmission. Further, we evaluate the use of ONT long reads to detect lineage-specific structural variants, including in highly variable gene regions such as *pe/ppe* genes, which are potential vaccine targets. Overall, our work reinforces the utility of WGS of *M. tuberculosis* to reveal DR mutations for clinical management, and provides evidence of transmission for surveillance activities, thereby assisting TB control and elimination efforts.

## Results

### Whole genome sequencing and genomic variants

Fifty-nine paired *M. tuberculosis* DNA sourced from 5 regions across Thailand (Table 1; Table S1) were sequenced on Illumina and ONT platforms and underwent bioinformatic analysis (see Fig. S1 for pipelines). Across the paired samples, the mean read lengths for Illumina were 149bp and ONT were 2115bp (interquartile range (IQR): 655–2594 bp). The mean number of reads for Illumina was 2,599,368 (range: 707,135—4,174,158) and for ONT was 42,596 (range: 6572–121,132). The alignment of the sequence data to the H37rv reference genome revealed differences in the average depth between platforms (Illumina 90.2-fold, ONT 17.4-fold), but the percentage of the genome covered to at least five-fold was high across both technologies (ONT 98.9%, Illumina 98.7%). The average coverage of genes across ONT and Illumina replicates was correlated (Spearman’s *rho* = 0.30), including across DR (*rho* = 0.25), *pe/ppe* (*rho* = 0.57), and other loci (*rho* = 0.29) (Fig. S2). Average coverage was lower (< 60-fold) in regions with any lineage-specific large deletions, including within a cluster of genes encompassing *rv1573* to *rv1586* (deletion in lineage 2), and *ppe50* (deletion in lineage 1) (Figs. S2, S3). The average number of high-quality SNPs (see “Methods”) for Illumina was 1618 (range: 561–2012) and for ONT was 1567 (range: 548–2095). Between DNA replicates and platforms, the number of paired differences was low (median 18, IQR 8–55) and SNP ratios were close to 1 (Illumina to ONT ratio: median 1.002, IQR 0.987–1.016) (Table S2).

**Table 1:** Characteristics of the 59 *M. tuberculosis* isolates.

Characteristic	Median (N)	Range (%)
Age (years)	59	24–82
Gender (male)	39	66.1
Location		
Chiang Rai	27	45.7
Kalasin	2	3.4
Khon Kaen	17	28.8
Maha Sarakham	8	13.6
Roi Et	5	8.4
Lineage		
1	30	50.8
2	25	42.3
3	1	1.7
4	3	5.1
Drug resistance*		
Any	30	50.8
Rifampicin**	7	11.8
Isoniazid**	15	25.4
MDR-TB	6	10.1
Pre-XDR	1	1.7

*Genotypic resistance; **matches the phenotypic drug susceptibility test results.

*MDR* multidrug resistant, *XDR* extensively drug resistant.

### Lineages

Using TB-Profiler software, the lineage and genotypic resistance profiles were identical between the ONT and Illumina pairs. The majority of isolates were from lineages 1 (L1 n = 30, 50.8%) and 2 (L2 n = 25, 42.3%), with a minority for lineages 3 (L3 n = 1, 1.7%) and 4 (L4 n = 3, 5.1%) (Table 1; Table S1), which is broadly representative of the prevalence in Thailand^5,6^. The ONT data allowed for the identification of lineage-specific large deletions (Regions of Difference (RDs)). Beijing strains (L2) possessed RD105, whilst RD239 and RD147c were unique to L1, RD750 for L3, and RD122 for L4. The RD11 deletion was present in lineage L1.1.1, and absent in L1.2.1.2.1 (20/59), aligning with previous studies^7^. RD210 was universally detected in lineage L1.2.1.2.1 strains (8/59). RD152 was present in all L2.2 and L3 strains, supporting findings from other studies^8^ (Table S3).

### Drug resistance

Thirty isolates (50.1%) were resistant to any anti TB drug, including for isoniazid (15/59, 25.4%), rifampicin (7/59, 11.8%), MDR-TB (5/59, 8.4%), and pre-XDR (1/59, 1.7%) (Table 1). Phenotypic drug susceptibility data was generated for rifampicin and isoniazid, and confirmed the genotypic resistance profiles. The frequencies of DR mutations were compared to those from public Thailand (“other Thailand”, n = 1456) and “Global50k” (n = 50,722) datasets (Table 2; Table S1). The most frequent mutations that underly isoniazid resistance were *katG* Ser315Thr (9/59; other Thailand 56.9%; Global 78.6%) and *fabG1* -15C>T (5/59; other Thailand 6.1%; Global 25.0%). For rifampicin, the *rpoB* 450 codon mutations Ser450Trp and Ser450Leu were present (5/59; other Thailand 36.4%; Global 58.8%). There were some mutations identified that are considered less prevalent, namely for ethionamide (*ethA* Thr232Ala 5/59) and pyrazinamide (*pncA* Ser104Arg 5/59). The underlying mutations for ciprofloxacin or fluoroquinolone resistance were *gyrA* Asp94Gly (1/59) and *gyrB* Asp494Ala (1/59). The PAS-linked *thyX* -16C>T mutation was also detected (1/59). Several potential streptomycin mutations were also found (*rpsL* Lys43Arg, *rpsL* Lys88Arg, *rrs* 514A>C, *rrs* 517C>T) (Table 2).

**Table 2:** Drug resistance mutations identified in the 59 *M. tuberculosis* isolates.

Drug	Gene	Mutation**	Our study N	Our study %	Other Thailand %	Global %
			(/59)		(n = 1456)***	(n = 50k)****
Isoniazid	*fabG1*	-15C>T	5	8.4	6.1	25.0
Isoniazid	*katG*	Ser315Thr	9	15.3	56.9	78.6
Isoniazid	*katG*	1002_1003dupGA	1	1.7	0.1	0.0
Rifampicin	*rpoB*	Ser450Trp	2	3.4	0.8	1.1
Rifampicin	*rpoB*	His445Tyr	2	3.4	7.5	3.3
Rifampicin	*rpoB*	Ser450Leu	3	5.1	35.6	57.7
Ethionamide	*ethA*	Thr232Ala	5	8.4	0.5	0.0
Ethionamide	*fabG1*	-15C>T	5	8.4	6.1	25.0
Ethambutol	*embA*	-16C>T	1	1.7	0.8	2.4
Ethambutol	*embB*	Arg507Lys	1	1.7	0.1	0.0
Ethambutol	*embB*	Met306Leu	1	1.7	1.0	1.2
Pyrazinamide	*pncA*	Ser104Arg	5	8.4	0.7	0.5
Pyrazinamide	*pncA*	Thr142Lys	1	1.7	0.2	0.1
Ciprofloxacin	*gyrA*	Asp94Gly	1	1.7	5.1	11.7
Ciprofloxacin	*gyrB*	Asp494Ala	1	1.7	0.6	0.0
Fluoroquinolones	*gyrA*	Asp94Gly	1	1.7	5.1	11.7
Fluoroquinolones	*gyrB*	Asp494Ala	1	1.7	0.6	0.0
PAS	*thyX*	-16C>T	1	1.7	2.5	2.4
Streptomycin	*gid*	Glu92*	1	1.7	0.1	0.1
Streptomycin	*gid*	351delG	1	1.7	1.0	1.8
Streptomycin	*gid*	351dupG	1	1.7	0.3	0.4
Streptomycin	*rpsL*	Lys43Arg	3	5.1	37.6	38.9
Streptomycin	*rpsL*	Lys88Arg	2	3.4	3.6	9.9
Streptomycin	*rrs*	514A>C	2	3	1.6	7.3
Streptomycin	*rrs*	517C>T	1	1.7	0.8	4.6

*stop codon; **supported by Oxford Nanopore Technology (ONT) (whole genome) and Illumina sequencing; PAS para-aminosalicylic acid; ***from^2,6^; ****from^2^.

Using the ONT data, we scanned for structural variants across known drug-resistant genes (Table 3). Deletions were identified in 10 isolates, and confirmed by Illumina data. The size of deletions identified were similar across platforms (range: ONT 35–10,981 bp; Illumina 27–10,983 bp), with those in *Rv3083, Rv1258c*, *eis* and *thyA* covering almost their entire genes. One isolate (S27) had a deleted *eis* gene. It has been found that overexpression of *eis* is a leading cause of resistance to the antibiotic kanamycin, and its complete deletion could enhance the *M. tuberculosis* susceptibility to antibiotics, an inversion of the typical DR mechanism^9^. Additionally, we identified several isolates with deletions towards the end of the *fbiC* gene, which may influence the effectiveness of certain antibiotics^10^. Some studies have indicated a link between mutations occurring near the end of the gene and an increase in the bacteria's MIC and heightened resistance to delamanid^11^. These findings underscore the need for further research into the implications of these *fbiC* deletions.

**Table 3:** Deletions found using the Oxford Nanopore Technology platform.

Sample	Drug resistance profile	Gene*
S13	Streptomycin, Capreomycin, Amikacin	*whiB6*
S14	Ethionamide	Rv3083**
S22	Streptomycin, Pyrazinamide, Isoniazid	Rv1258c**
S27	Kanamycin, Amikacin, PAS	eis, thyA**
S37	Streptomycin	*gid*
S49	Ethambutol	*embR*
S50	PAS	thyA**
S66	Delamanid	*fbiC*
S67	Delamanid	*fbiC*
S68	Ethambutol	*embR*

*Confirmed using Illumina data; PAS Para-aminosalicylic acid; ** > 95% of gene deleted.

### Phylogenetic analysis and transmission

**Figure 1:**
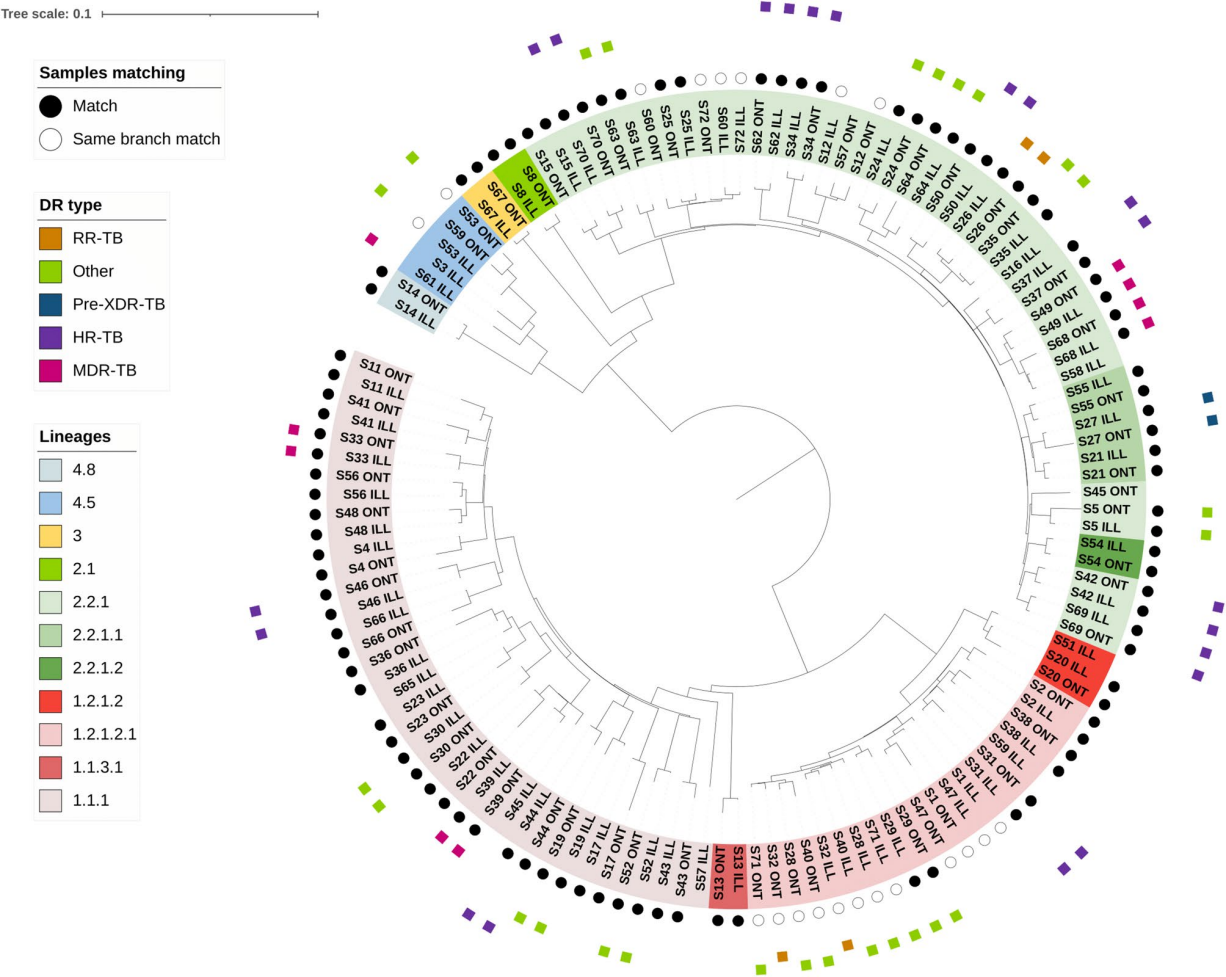
Phylogenetic tree analysis. Phylogenetic tree reveals high degree of concordance and clustering of replicates sequenced using Oxford Nanopore Technologies (ONT) and Illumina platforms.

**Figure 2:**
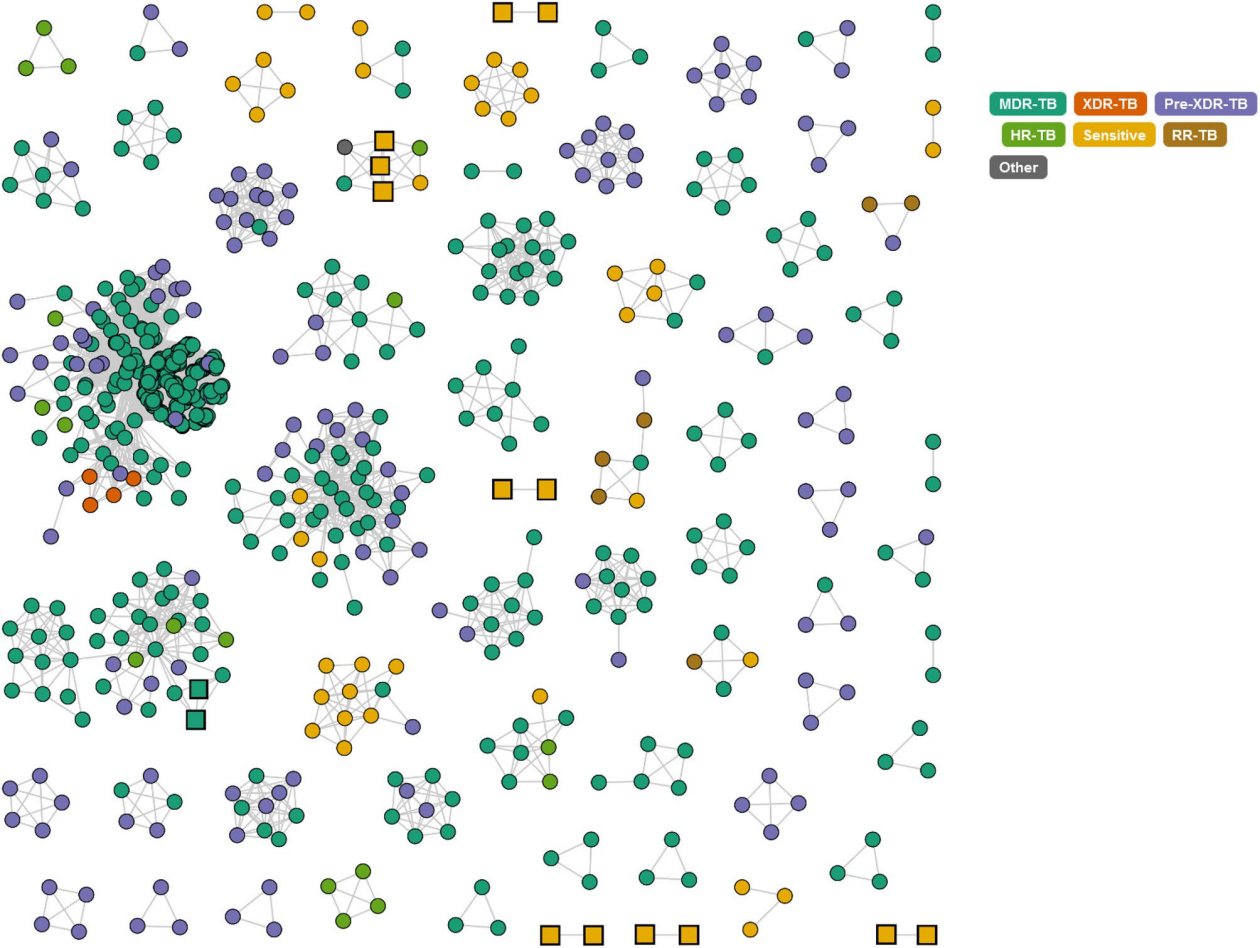
Combined Thailand analysis of our study isolates (n = 59; squares) and other Thailand (n = 1456; circles) isolates reveals clusters of high similarity. We applied a cut-off value of 20 SNPs or less differences between linked isolates. Squares are samples included in this study, and the image was created using the “tgv” tool (https://github.com/jodyphelan/tgv).

Using SNPs detected across all samples, a phylogenetic analysis revealed the expected clustering by lineage, and that replicate isolates were paired together on the tree, indicating minimal divergence between ONT and Illumina-sequenced isolates. However, ONT-sequenced isolates tend to demonstrate marginally elongated branch lengths (Fig. 1). In a combined analysis with the other Thailand isolates (n = 1456), at a SNP similarity threshold of at most 20 SNPs difference, 153 clusters were identified, with sizes ranging from 2 to 224, and a median size of 2 (Fig. 2). Seven clusters include 15 study isolates, with five clusters containing exclusively two study isolates each. A further cluster (n = 41) contained two study isolates and was formed of at least MDR-TB samples sourced from the Roi Et region. The other cluster (n = 7) had 3 study isolates with drug sensitivity (all from Chiang Rai). At thresholds of 5 and 10 SNPs differences, there were 134 (size range: 2–150 samples) and 154 (size range: 2–213 samples) clusters, respectively. At these more stringent SNP thresholds there were at least 17 study isolates within clusters of sizes 2 or more, including two of our study isolates within the MDR-TB dominated clade.

### Structural variants in *pe/ppe* genes

**Figure 3:**
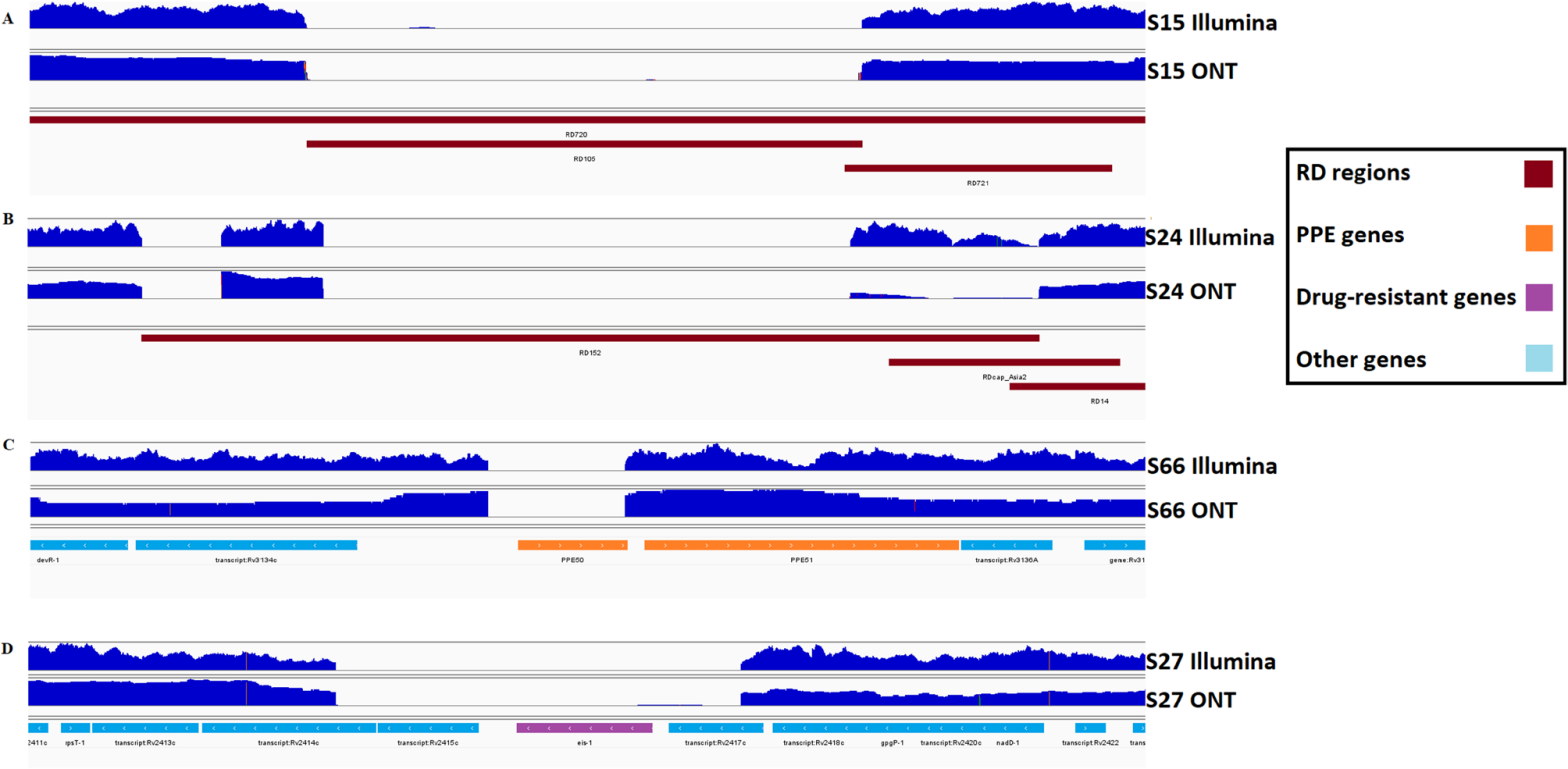
Sequence coverage plots showing selected detected structural variants related to drug resistance and lineages (regions of difference, RD) identified in Oxford Nanopore Technologies (ONT) and Illumina (Illumina) platforms. Coverage is indicated by blue mounds with isolate labels on the side. (**A**) RD105 deletion confirmed in isolate S15 (lineage 2.2.1). All samples from lineage 2 contained this deletion; (**B**) RD152 deletion confirmed in isolate S24. While it does not span the entire deletion, a consistent pattern has been observed across all samples from lineage 2, suggesting shared deletion characteristics; (**C**) a deletion in the *ppe50* gene in isolate S66 that was present across all lineage 1 strains; (**D**) an entire deletion of the *eis* gene in isolate S27, potentially linked to drug resistance.

*Pe/ppe* genes are highly variable gene regions within the *M. tuberculosis* genome (~ 10%) that are implicated in interactions with the human host^12^. Due to their highly variable nature, they are typically removed from analysis. However, using ONT sequencing data, we identified 830 deletions in 56 *pe/ppe* genes across all samples (Table S4). The analysis revealed several lineage-specific deletions. For example, the complete deletion of the *ppe50* gene in L1 strains (Fig. 3), the *ppe66/67* deletion in L3 strains (Table S4), and small deletions on *pe_pgrs2* and *pe_pgrs6* which were L1.2.1.2.1 specific. There was complexity in the *ppe8* gene, with deletions at the gene start for L1 strains and the encompassed RD304 region for L2. There were also gene fusion events, where all L2 strains had *pe_pgrs3* and *pe_pgrs4* gene deletions at their respective gene boundaries. Several *pe/ppe* genes, including *ppe34* and *pe_pgrs10*, were found to have small deletions across most of the study isolates. Our lineage-specific structural variants corroborated earlier studies that used PacBio long read data^12^ (Table S4), and underscore the reliability and reproducibility of our approach and data.

## Discussion

There are increasingly cited benefits of using whole-genome sequencing (WGS) technologies in clinical and epidemiological settings, including through the characterisation of transmission networks, or for the detection of drug-resistance (DR) associated mutations to inform on “precision medicine” treatment decisions^13^. The direct sequencing from sputum samples has been reported, taking less than a week, which will shorten the time from specimen collection to a DR profile, leading to timely and personalised treatment that can be significantly delayed when culture isolation is required (up to 3 weeks). With WGS approaches gaining traction, ONT and other rapid platforms are expected to play a key role in clinical and surveillance settings, especially in those regions with a high TB burden, such as Southeast Asia.

Although, ONT is known to have a higher error rate, compared to other sequencing platforms, previous work has shown it is possible to robustly call SNPs^4^. In our study, we performed a paired sequencing analysis of 59 *M. tuberculosis* replicate DNA sourced from Thailand, and compared WGS data from ONT and Illumina platforms. Our analysis revealed a high concordance in variant calls and positioning on a phylogenetic tree. A combined analysis with publicly available Thailand *M. tuberculosis* WGS (n = 1456) revealed that a subset of our 59 study isolates have high genomic similarity to those nationwide, indicative of their role in transmission. The high concordance between ONT and Illumina platforms, suggests that relatively higher ONT error rates are not prohibitive for diagnostic applications in the clinic. Further, the use of ONT long reads provided insights into strain-specific structural variants, including in highly variable gene regions such as *pe/ppe* genes, which are potential vaccine targets. The use of long reads can cover repetitive regions of the genome, and thereby help elucidate compensatory or epistatic mutations that could be crucial for the better understanding of DR mechanisms in *M. tuberculosis*, as well as *pe/ppe* genes that have been linked to host immunity and thereby vaccine targets. For a more comprehensive analysis, it is important to incorporate samples from all lineages within the *Mycobacterium tuberculosis complex*, as well as other omics (e.g., RNA-seq). Our current investigation predominantly focuses on L1 and L2, which are dominant in Thailand.

In conclusion, we have shown that ONT data is useful for epidemiological, phylogenetic, or drug resistance detection applications, and therefore can provide much needed assistance in the control of TB, especially in high burden settings (e.g., Thailand) where impacts will be greater.

## Methods

### Culture, DNA extraction and sequencing

The 59 isolates analysed in this study were sourced from TB patients across 5 regions (Thailand), and chosen randomly from Thailand Ministry of Public Health (MOPH) stored samples, spanning years 2020 and 2022. DNA extraction was performed by using Presto™ Mini gDNA Bacteria Kit (Cat. GBB300/301, Geneaid Biotech Ltd., Taiwan) with some modifications in the sample preparation process. Briefly, the *M. tuberculosis* colonies from solid cultures (Lowenstein-Jensen media) were resuspended in 500–750 µl of PBS with glass beads and vigorously mixed by vortex to make homogeneous mycobacterial suspensions before heat inactivated at 80 °C for 20 min. A total of 200 µl of the suspensions were used for DNA extraction following the manufacturer’s protocol with RNase A treatment. The final volume of the elution was 60 µl. The WGS of DNA samples was performed with ONT (MinION Flow Cell with R10.4 with Kit 12 chemistry; SQK-NBD112.24 ligation-based sequencing kit) and Illumina NextSeq 500/550 (Mid Output Kit v2.5; 300 Cycles) using Illumina DNA Prep and IDT® for Illumina® (DNA/RNA UD Indexes Set B) for library preparation. Drug susceptibility testing was performed as part of routine TB culture and phenotypic assessments for rifampicin and isoniazid at the Thailand MOPH, using established protocols (see^7^). All laboratory work was performed in Thailand, in accordance with relevant guidelines and regulations.

### Bioinformatics pipeline

Base calling of ONT raw sequence data was performed with the bonito basecaller (model dna_r9.4.1_e8.1_sup@v3.3) and non-ambiguous reads were aligned to the H37Rv reference genome (GCA_000195955.2) using minimap2 (v2.24) software^14^. The depth of coverage along the genome and per gene was calculated with BEDTools (v2.29.2)^15^, using the alignments of data obtained by ONT and Illumina platforms. To compare between samples, median coverage per gene per isolate was normalised by the coverage of four housekeeping genes (*gyrB*, *gyrA*, *rpoB*, *rpoC*), known not to be deleted or duplicated and expected to have a good “average” coverage. Variant calling of SNPs and small indels was carried out using Freebayes (v1.3.5) software^16^, filtering the outputs with a read depth of at least five-fold, and a genotype (GT) parameter of 1, leading to only high quality SNPs. Delly software (v0.8.7)^17^ was used to call large structural variants (SVs; indels with size > 15 bp) for both the 59 Illumina and ONT samples. Sniffles (v2.0.7) software^17^ was used to confirm ONT findings. These large SVs were then visualised in IGV^19^ to confirm the variants across all *pe/ppe* genes, RDs and DR linked loci. Lineage and DR profiling of the sample pairs was carried out with TB-Profiler (v4.4.2)^3^. Maximum likelihood phylogenetic reconstruction of the genomes was performed with IQ-TREE (v2.2.0.3) (model: TVM + F + ASC nucleotide substitution)^20^ using genome-wide SNPs, and visualised together with annotations in iTOL software. Any annotations used for drug resistant discovery or *pe/ppe* genes analysis was made with snpEff (v5.1) software^21^. The Thai isolates were compared to other samples with public WGS from the same country (n = 1456; see^2,6^) and sourced globally (n = 50,722;^2^). To obtain the transmission graph, a combined variant call format (VCF) file for the entire Thailand dataset was generated, and a between isolate distance matrix was inferred and formatted for application by the “tgv” webtool (https://github.com/jodyphelan/tgv). Potential transmission clusters were constructed using cut-offs of 20, 10 and 5 SNP differences between isolates, with the higher threshold preferred after evaluating the tail of the distribution of pairwise differences^22^. Whilst previous work in a high TB incidence area used a cut-off of 10 SNPs, this approach can be over-simplistic and affected by several factors (e.g., culturing protocol and bioinformatics pipelines), so we present results from a range of difference values^22^. All scripts used in the analysis pipeline (see Fig. S1) are available in a GitHub repository (https://github.com/klausyboi/ont-illumina-comparison-data).

### Ethics approval and consent

The studies were approved by the Thailand Ministry of Public Health ethics committee. Informed written consent was sought and obtained for all patients in the original study.

### Supplementary Information

Supplementary Information.

## Data Availability

Raw sequencing data is available from the ENA archive (see Table S1 for a list of accession numbers).

## References

[1] World Health Organization (2022). Global tuberculosis report 2022.

[2] Napier, G. *et al.* Robust barcoding and identification of mycobacterium tuberculosis lineages for epidemiological and clinical studies. *Genome Med.* **12,** (2020). 10.1186/s13073-020-00817-3 PMC7734807 33317631

[3] Phelan, J. E. *et al.* Integrating Informatics Tools and portable sequencing technology for rapid detection of resistance to anti-tuberculous drugs. *Genome Med.* **11** (2019). 10.1186/s13073-019-0650-x PMC6591855 31234910

[4] Gómez-González, P. J., Campino, S., Phelan, J. E. & Clark, T. G. Portable sequencing of *mycobacterium tuberculosis* for clinical and epidemiological applications. *Brief. Bioinf.* **23** (2022). 10.1093/bib/bbac256 PMC9487601 35894606

[5] O’Neill MB (2019). Lineage specific histories of *mycobacterium tuberculosis* dispersal in Africa and Eurasia. Mol. Ecol..

[6] Phelan, J. *et al.* Genome-wide host-pathogen analyses reveal genetic interaction points in tuberculosis disease. *Nat. Commun.* **14,** (2023). 10.1038/s41467-023-36282-w PMC9892022 36725857

[7] Coker, O. O. *et al.* Genetic signatures of *mycobacterium tuberculosis* nonthaburi genotype revealed by whole genome analysis of isolates from tuberculous meningitis patients in Thailand. *PeerJ* **4,** (2016). 10.7717/peerj.1905 PMC4841212 27114869

[8] Kanji A, Hasan Z, Tanveer M, Laiq R, Hasan R (2011). Occurrence of RD149 and RD152 deletions in mycobacterium tuberculosis strains from Pakistan. J. Infect. Dev. Count..

[9] Sowajassatakul, A., Prammananan, T., Chaiprasert, A. & Phunpruch, S. Overexpression of EIS without a mutation in promoter region of amikacin- and kanamycin-resistant mycobacterium tuberculosis clinical strain. *Ann. Clin. Microbiol. Antimicrob. ***17,** (2018). 10.1186/s12941-018-0285-6 PMC6047124 30008266

[10] Rifat, D. *et al.* Mutations in *fbid* (*rv2983*) as a novel determinant of resistance to pretomanid and Delamanid in mycobacterium tuberculosis. *Antimicrob. Agents Chemother.* **65** (2020). 10.1128/AAC.01948-20 PMC7927868 33077652

[11] Battaglia, S. *et al.* Characterization of genomic variants associated with resistance to Bedaquiline and delamanid in naive mycobacterium tuberculosis clinical strains. *J. Clin. Microbiol.* **58,** (2020). 10.1128/JCM.01304-20 PMC7587096 32907992

[12] Gómez-González, P. J. *et al.* Functional genetic variation in PE/PPE genes contributes to diversity in mycobacterium tuberculosis lineages and potential interactions with the human host. *Front. Microbiol.* **14**, (2023). 10.3389/fmicb.2023.1244319 PMC10591178 37876785

[13] Yam, E. L. *et al.* Antimicrobial resistance in the Asia Pacific Region: A meeting report. *Antimicrob. Resist Infect. Control* **8,** (2019). 10.1186/s13756-019-0654-8 PMC6921568 31890158

[14] Li H (2018). Minimap2: Pairwise alignment for nucleotide sequences. Bioinformatics.

[15] Quinlan AR, Hall IM (2010). BEDTools: A flexible suite of utilities for comparing genomic features. Bioinformatics.

[16] Garrison, E. & Marth, G. Haplotype-based variant detection from short-read sequencing. arXiv:1207 (2012)

[17] Rausch T (2012). Delly: Structural variant discovery by integrated paired-end and split-read analysis. Bioinformatics.

[18] Sedlazeck FJ (2018). Accurate detection of complex structural variations using single-molecule sequencing. Nat. Methods.

[19] Robinson, J. T., Thorvaldsdóttir, H., Wenger, A. M., Zehir, A. & Mesirov, J. P. Variant review with the integrative genomics viewer. *Cancer Res.* **77** (2017). 10.1158/0008-5472.CAN-17-0337 PMC5678989 29092934

[20] Nguyen L-T, Schmidt HA, von Haeseler A, Minh BQ (2014). IQ-tree: A fast and effective stochastic algorithm for estimating maximum-likelihood phylogenies. Mol. Biol. Evol..

[21] Cingolani P (2012). A program for annotating and predicting the effects of single nucleotide polymorphisms. SnpEff. Fly.

[22] Sobkowiak B, Banda L, Mzembe T, Crampin AC, Glynn JR, Clark TG (2020). Bayesian reconstruction of *Mycobacterium tuberculosis* transmission networks in a high incidence area over two decades in Malawi reveals associated risk factors and genomic variants. Microb. Genom..

