# Supplementary material for: Multi-platform whole genome sequencing for tuberculosis clinical and surveillance applications
Source: Sci Rep. 2024 Mar 3;14:5201. doi: 10.1038/s41598-024-55865-1 (PMC10908857; doi:10.1038/s41598-024-55865-1)
Supplement: Supplementary file 1 — Supplementary Information. [file 41598_2024_55865_MOESM1_ESM.pdf]

**Table S1**

**Study isolates undergoing sequencing using Illumina and Oxford Nanopore Technology (ONT) platforms (N=59)**

| Sample | Illumina Accession no. | ONT accession no. | Lineage* | Drug Resistance** | Drug resistance Mutations** |
|---|---|---|---|---|---|
| **S1** | ERR12328206 | ERR12328582 | 1.2.1.2.1 | Sensitive | |
| **S2** | ERR12328205 | ERR12328581 | 1.2.1.2.1 | Sensitive | |
| **S4** | ERR12328204 | ERR12328580 | 1.1.1 | Sensitive | |
| **S5** | ERR12328203 | ERR12328579 | 2.2.1 | Other | Streptomycin *gid* 351delG |
| **S8** | ERR12328202 | ERR12328578 | 2.1 | Sensitive | |
| **S11** | ERR12328201 | ERR12328577 | 1.1.1 | Sensitive | |
| **S12** | ERR12328200 | ERR12328576 | 2.2.1 | Sensitive | |
| **S13** | ERR12328199 | ERR12328575 | 1.1.3.1 | Sensitive | |
| **S14** | ERR12328198 | ERR12328574 | 4.8 | Sensitive | |
| **S15** | ERR12328197 | ERR12328573 | 2.2.1 | Sensitive | |
| **S17** | ERR12328196 | ERR12328572 | 1.1.1 | Sensitive | |
| **S19** | ERR12328195 | ERR12328571 | 1.1.1.1 | Other | Streptomycin *gid* Glu92 |
| **S20** | ERR12328194 | ERR12328570 | 1.2.1.2 | Sensitive | |
| **S21** | ERR12328193 | ERR12328569 | 2.2.1.1 | Sensitive | |
| **S22** | ERR12328192 | ERR12328568 | 1.1.1 | Sensitive | |
| **S23** | ERR12328191 | ERR12328567 | 1.1.1 | Sensitive | |
| **S24** | ERR12328190 | ERR12328566 | 2.2.1 | Other | FQs *gyrB* Asp494Ala *** |
| **S25** | ERR12328189 | ERR12328565 | 2.2.1 | Sensitive | |
| **S26** | ERR12328188 | ERR12328564 | 2.2.1 | RR-TB | *rpoB* Ser450Trp |
| **S27** | ERR12328187 | ERR12328563 | 2.2.1.1 | Pre-XDR-TB | Rifampicin *rpoB* Ser450Trp; Isoniazid *katG* Ser315Thr; Ethambutol *embA* -16C>T; PAS *thyX* -16C>T; Streptomycin *rpsL* Lys43Arg; FQs *gyrA* Asp94Gly |
| **S28** | ERR12328186 | ERR12328562 | 1.2.1.2.1 | Other | Pyrazinamide *pncA* Ser104Arg; Ethionamide *ethA* Thr232Ala |
| **S29** | ERR12328185 | ERR12328561 | 1.2.1.2.1 | Other | Pyrazinamide *pncA* Ser104Arg; Ethionamide *ethA* Thr232Ala |
| **S30** | ERR12328184 | ERR12328560 | 1.1.1 | Other | Pyrazinamide *pncA* Thr142Lys; |
| **S31** | ERR12328183 | ERR12328559 | 1.2.1.2.1 | HR-TB | Isoniazid *fabG1* -15C>T; Streptomycin *rpsL* Lys88Arg; Ethionamide *fabG1* -15C>T; Streptomycin *gid* c.351dupG |
| **S32** | ERR12328182 | ERR12328558 | 1.2.1.2.1 | RR-TB | Pyrazinamide *pncA* Ser104Arg; Ethionamide *ethA* Thr232Ala; Rifampicin *rpoB* Ser450Leu |
| **S33** | ERR12328181 | ERR12328557 | 1.1.1 | MDR-TB | Isoniazid *fabG1* -15C>T; Ethionamide *fabG1* -15C>T; Rifampicin *rpoB* Ser450Leu |
| **S34** | ERR12328180 | ERR12328556 | 2.2.1 | HR-TB | Isoniazid *katG* Ser315Thr; |
| **S35** | ERR12328179 | ERR12328555 | 2.2.1 | Other | Streptomycin *rrs* 514A>C |
| **S36** | ERR12328178 | ERR12328554 | 1.1.1 | Sensitive | |
| **S37** | ERR12328177 | ERR12328553 | 2.2.1 | HR-TB | Isoniazid *katG* Ser315Thr; |

| | | | | | |
|---|---|---|---|---|---|
| **S38** | ERR12328176 | ERR12328552 | 1.2.1.2.1, 1.1.1 | Sensitive | |
| **S39** | ERR12328175 | ERR12328551 | 1.1.1 | MDR-TB | Isoniazid *fabG1* -15C>T; Ethionamide *fabG1* -15C>T; Rifampicin *rpoB* His445Tyr |
| **S40** | ERR12328174 | ERR12328550 | 1.2.1.2.1 | Other | Pyrazinamide *pncA* Ser104Arg; Ethionamide *ethA* Thr232Ala |
| **S41** | ERR12328173 | ERR12328549 | 1.1.1 | Sensitive | |
| **S42** | ERR12328172 | ERR12328548 | 2.2.1,1.1.3.3 | HR-TB | Isoniazid *katG* Ser315Thr |
| **S43** | ERR12328171 | ERR12328547 | 1.1.1 | Sensitive | |
| **S44** | ERR12328170 | ERR12328546 | 1.1.1 | HR-TB | Isoniazid *fabG1* -15C>T; Ethionamide *fabG1* -15C>T |
| **S46** | ERR12328168 | ERR12328544 | 1.1.1 | HR-TB | Isoniazid *katG* Ser315Thr |
| **S47** | ERR12328167 | ERR12328543 | 1.2.1.2.1 | Sensitive | |
| **S48** | ERR12328166 | ERR12328542 | 1.1.1 | Sensitive | |
| **S49** | ERR12328165 | ERR12328541 | 2.2.1 | MDR-TB | Streptomycin *rpsL* Lys43Arg; Ethambutol *embB* Met306Leu; Rifampicin *rpoB* Ser450Leu; Isoniazid *katG* Ser315Thr |
| **S50** | ERR12328164 | ERR12328540 | 2.2.1 | HR-TB | Streptomycin *rpsL* Lys88Arg; Isoniazid *katG* Ser315Thr |
| **S52** | ERR12328163 | ERR12328539 | 1.1.1 | Other | Ethambutol *embB* Arg507Lys |
| **S53** | ERR12328162 | ERR12328538 | 4.5 | Sensitive | |
| **S54** | ERR12328161 | ERR12328537 | 2.2.1.2 | Sensitive | |
| **S55** | ERR12328160 | ERR12328536 | 2.2.1.1 | Sensitive | |
| **S56** | ERR12328159 | ERR12328535 | 1.1.1 | Sensitive | |
| **S58** | ERR12328158 | ERR12328534 | 2.2.1 | MDR-TB | Rifampicin *rpoB* His445Asp; *katG* Ser315.Thr |
| **S60** | ERR12328157 | ERR12328533 | 2.2.1 | Sensitive | |
| **S62** | ERR12328156 | ERR12328532 | 2.2.1 | HR-TB | Isoniazid *katG* Ser315Thr |
| **S63** | ERR12328155 | ERR12328531 | 2.2.1 | Other | Streptomycin rrs 514A>C |
| **S64** | ERR12328154 | ERR12328530 | 4.2,2.2 | Other | Streptomycin rrs 517C>T |
| **S66** | ERR12328153 | ERR12328529 | 1.1.1 | Sensitive | |
| **S67** | ERR12328152 | ERR12328528 | 3 | Sensitive | |
| **S68** | ERR12328151 | ERR12328527 | 2.2.1 | MDR-TB | Rifampicin *rpoB* His445Tyr; Isoniazid *katG* Ser315Thr |
| **S69** | ERR12328150 | ERR12328526 | 2.2.1 | HR-TB | Isoniazid *katG* 1002_1003dupGA; Streptomycin *rpsL* Lys43Arg |
| **S70** | ERR12328149 | ERR12328525 | 2.2.1 | HR-TB | Isoniazid *fabG1* -15C>T; Ethionamide *fabG1* -15C>T |
| **S71** | ERR12328148 | ERR12328524 | 1.2.1.2.1 | Other | Pyrazinamide *pncA* Ser104Arg; Ethionamide *ethA* Thr232Ala |
| **S72** | ERR12328147 | ERR12328523 | 2.2.1 | Sensitive | |

* based on TB-Profiler, and matched identically between ONT and Illumina; ** based on TB-Profiler, and drug resistance type and mutations matched identically between platforms; HR-TB Isoniazid drug resistant; MDR-TB multidrug resistant TB; RR-TB rifampicin resistant TB, Fluoroquinolones (FQs): Moxifloxacin, Ofloxacin, Levofloxacin, Ciprofloxacin

**Table S2**

**Paired isolates (n=59) sequenced using Illumina and Oxford Nanopore Technology (ONT) platforms**

| Sample | Platform | Ave. Depth | No. SNPS* | % Mapped | Total Reads |
|---|---|---|---|---|---|
| **S1** | ONT | 5.4 | 1168 | 99.6% | 6572 |
| | Illumina | 103.2 | 1954 | 98.8% | 2963742 |
| **S2** | ONT | 13.5 | 1934 | 99.4% | 19654 |
| | Illumina | 41.4 | 1944 | 98.8% | 1176138 |
| **S4** | ONT | 9.9 | 1923 | 99.6% | 17861 |
| | Illumina | 109.1 | 1985 | 98.8% | 3137078 |
| **S5** | ONT | 6.1 | 985 | 99.8% | 10075 |
| | Illumina | 78.3 | 1345 | 99.0% | 2217701 |
| **S8** | ONT | 22.9 | 1322 | 100% | 43766 |
| | Illumina | 73.2 | 1273 | 99.4% | 2300179 |
| **S11** | ONT | 38.1 | 2095 | 99.2% | 65534 |
| | Illumina | 100.1 | 1963 | 98.7% | 2873142 |
| **S12** | ONT | 5.9 | 921 | 99.7% | 15899 |
| | Illumina | 76.1 | 1376 | 99.0% | 2163404 |
| **S13** | ONT | 9.2 | 1888 | 99.6% | 17150 |
| | Illumina | 98.9 | 2012 | 98.8% | 2837108 |
| **S14** | ONT | 31.7 | 548 | 99.8% | 21618 |
| | Illumina | 135.5 | 561 | 99.6% | 3912222 |
| **S15** | ONT | 23.9 | 1353 | 96.2% | 28957 |
| | Illumina | 89.6 | 1313 | 98.9% | 2561666 |
| **S17** | ONT | 11.7 | 1990 | 99.8% | 18354 |
| | Illumina | 112.9 | 1952 | 98.9% | 3260628 |
| **S19** | ONT | 15.2 | 1953 | 99.8% | 22559 |
| | Illumina | 74.8 | 1971 | 98.8% | 2121102 |
| **S20** | ONT | 10.2 | 1856 | 99.7% | 24968 |
| | Illumina | 76.1 | 1925 | 98.8% | 2164348 |
| **S21** | ONT | 18.1 | 1304 | 99.6% | 32668 |
| | Illumina | 92.2 | 1316 | 99.0% | 2634471 |
| **S22** | ONT | 10.7 | 1963 | 99.7% | 9765 |
| | Illumina | 121.3 | 1967 | 98.8% | 3506999 |
| **S23** | ONT | 27.1 | 2008 | 99.7% | 38927 |
| | Illumina | 112.1 | 1959 | 98.7% | 3227903 |
| **S24** | ONT | 30.4 | 1356 | 99.4% | 70506 |
| | Illumina | 28.0 | 1296 | 99.0% | 791827 |
| **S25** | ONT | 19.4 | 1263 | 92.7% | 70402 |
| | Illumina | 80.7 | 1249 | 94.8% | 2430535 |
| **S26** | ONT | 21.1 | 1321 | 99.8% | 67333 |
| | Illumina | 54.1 | 1318 | 99.1% | 1524118 |
| **S27** | ONT | 17.7 | 1508 | 88.9% | 50492 |
| | Illumina | 53.4 | 1316 | 99.1% | 1524609 |
| **S28** | ONT | 27.7 | 1955 | 99.6% | 63762 |
| | Illumina | 79.9 | 1952 | 98.7% | 2267204 |
| **S29** | ONT | 15.8 | 1947 | 99.6% | 42272 |

| | | | | | |
|---|---|---|---|---|---|
| | Illumina | 74.5 | 1943 | 98.5% | 2107027 |
| S30 | ONT | 15.1 | 1927 | 99.5% | 16899 |
| | Illumina | 105.8 | 1931 | 98.6% | 3018371 |
| S31 | ONT | 14.9 | 1947 | 99.6% | 19093 |
| | Illumina | 109.9 | 1932 | 98.8% | 3153821 |
| S32 | ONT | 24.7 | 1952 | 99.5% | 59494 |
| | Illumina | 52.6 | 1955 | 98.7% | 1487658 |
| S33 | ONT | 13.9 | 1991 | 99.6% | 16399 |
| | Illumina | 110.7 | 1958 | 98.8% | 3187457 |
| S34 | ONT | 16.0 | 1332 | 99.8% | 28349 |
| | Illumina | 108.4 | 1330 | 98.9% | 3076148 |
| S35 | ONT | 21.4 | 1330 | 99.6% | 99886 |
| | Illumina | 49.6 | 1313 | 98.9% | 1394378 |
| S36 | ONT | 8.0 | 1713 | 92.4% | 9976 |
| | Illumina | 139.3 | 1956 | 98.6% | 4019976 |
| S37 | ONT | 19.7 | 1311 | 99.7% | 89722 |
| | Illumina | 70.3 | 1319 | 99.1% | 2002450 |
| S38 | ONT | 22.5 | 1926 | 99.5% | 67398 |
| | Illumina | 74.4 | 1919 | 98.8% | 2141460 |
| S39 | ONT | 22.9 | 1958 | 99.2% | 78058 |
| | Illumina | 84.1 | 1950 | 98.6% | 2396636 |
| S40 | ONT | 17.2 | 1975 | 99.5% | 24331 |
| | Illumina | 110.0 | 1952 | 98.6% | 3138449 |
| S41 | ONT | 15.6 | 2000 | 99.6% | 27693 |
| | Illumina | 137.5 | 1976 | 98.7% | 3971276 |
| S42 | ONT | 16.8 | 1328 | 99.5% | 53347 |
| | Illumina | 25.1 | 1298 | 98.9% | 707135 |
| S43 | ONT | 28.9 | 1997 | 99.6% | 34487 |
| | Illumina | 143.5 | 1972 | 98.8% | 4174158 |
| S44 | ONT | 19.4 | 1922 | 99.5% | 34342 |
| | Illumina | 91.5 | 1919 | 98.7% | 2617699 |
| S46 | ONT | 21.5 | 1971 | 99.6% | 31625 |
| | Illumina | 115.6 | 1959 | 98.7% | 3309928 |
| S47 | ONT | 22.3 | 1948 | 99.4% | 31052 |
| | Illumina | 134.6 | 1958 | 98.8% | 3911533 |
| S48 | ONT | 22.1 | 1947 | 99.3% | 36838 |
| | Illumina | 111.0 | 1963 | 98.7% | 3190331 |
| S49 | ONT | 13.9 | 1323 | 99.7% | 52942 |
| | Illumina | 74.2 | 1327 | 99.0% | 2108101 |
| S50 | ONT | 21.0 | 1326 | 99.7% | 80478 |
| | Illumina | 48.7 | 1338 | 99.0% | 1373152 |
| S52 | ONT | 7.3 | 1625 | 99.5% | 8682 |
| | Illumina | 131.0 | 1972 | 98.6% | 3779393 |
| S53 | ONT | 6.0 | 556 | 99.6% | 9726 |
| | Illumina | 130.5 | 839 | 99.0% | 3780609 |
| S54 | ONT | 13.9 | 1336 | 99.6% | 27161 |
| | Illumina | 114.0 | 1335 | 98.9% | 3268429 |
| S55 | ONT | 15.6 | 1287 | 99.7% | 36221 |

| | | | | | |
|---|---|---|---|---|---|
| | Illumina | 110.9 | 1309 | 99.0% | 3176478 |
| **S56** | ONT | 19.1 | 1961 | 99.4% | 49455 |
| | Illumina | 128.7 | 1964 | 98.9% | 3731460 |
| **S58** | ONT | 7.0 | 1042 | 98.4% | 24233 |
| | Illumina | 106.1 | 1345 | 98.5% | 3029658 |
| **S60** | ONT | 16.3 | 1260 | 99.6% | 53081 |
| | Illumina | 83.3 | 1269 | 98.9% | 2366676 |
| **S62** | ONT | 28.0 | 1294 | 99.2% | 111928 |
| | Illumina | 60.8 | 1313 | 99.0% | 1726068 |
| **S63** | ONT | 14.4 | 1251 | 99.6% | 20474 |
| | Illumina | 116.8 | 1258 | 98.9% | 3344554 |
| **S64** | ONT | 9.0 | 1154 | 99.5% | 22348 |
| | Illumina | 89.3 | 1328 | 98.7% | 2534586 |
| **S66** | ONT | 6.6 | 1513 | 98.9% | 17966 |
| | Illumina | 33.2 | 1934 | 97.9% | 1513368 |
| **S67** | ONT | 21.8 | 1363 | 99.7% | 46238 |
| | Illumina | 120.8 | 1327 | 99.1% | 3475969 |
| **S68** | ONT | 23.9 | 1340 | 99.4% | 121132 |
| | Illumina | 68.6 | 1367 | 99.0% | 1949008 |
| **S69** | ONT | 20.3 | 1302 | 99.6% | 116354 |
| | Illumina | 39.2 | 1315 | 98.9% | 1099998 |
| **S70** | ONT | 12.7 | 1270 | 99.7% | 34644 |
| | Illumina | 75.8 | 1278 | 98.9% | 2140778 |
| **S71** | ONT | 17.4 | 1929 | 99.6% | 41764 |
| | Illumina | 105.5 | 1945 | 98.7% | 3017563 |
| **S72** | ONT | 22.0 | 1295 | 93.4% | 120256 |
| | Illumina | 47.7 | 1254 | 98.8% | 1344865 |

* High quality SNPs obtained at an alternate frequency of 0.7

** including low depth SNPs due to entire sample being lower than the cut-off (of 5)

**Table S3**

**Regions of difference (RD) deletions detected using the Oxford Nanopore Technology (ONT) platform**

| Sample | Lineage | RD* |
|---|---|---|
| S1 | 1.2.1.2.1 | RD147c, Rdbovis_wbbl2, RD715, RD239, RD210 |
| S2 | 1.2.1.2.1 | RD147c, Rdbovis_wbbl2, RD715, RD239, RD210, RD131e |
| S4 | 1.1.1 | RD715, RD147c, RDbovis_wbbl2, RD239, RD11, RD3, RD236a, RD131e, DS70 |
| S5 | 2.2.1 | RD105, RD3, RD152, RD715, RD206, MiD1 |
| S8 | 2.1 | RD105, RD715, RD720, RD721, RD152, RD314, N-RD25_tbB, RD131e |
| S11 | 1.1.1 | RD11 RD715, RD147c, RDbovis_wbbl2, RD3, RD236, RD239, RD131e, DS70 |
| S12 | 2.2.1 | RD105, RD181, RD311, RD3, RD181, RD311, RD3, RD207, RD715 |
| S13 | 1.1.3.1 | RD715, RD147c, RDbovis_wbbl2, RD239, RD11, RD131e, RD5sur |
| S14 | 4.8 | RD247b, RD715, MID4, RD219 |
| S15 | 2.2.1 | RD105, RD3, RD181, RD715, RD313, RD207, RD131e, RD152 |
| S17 | 1.1.1 | RD715, RD147c, RDbovis_wbbl2, RD239, RD11, RD3, RD236a, RD239 |
| S19 | 1.1.1.1 | RD715, RD147c, RDbovis_wbbl2, RD239, RD11, RD3, RD236a, RD239 |
| S20 | 1.2.1.2 | RD147c, Rdbovis_wbbl2, RD715, RD239, RD121, RD131e |
| S21 | 2.2.1.1 | RD105, RD715, RD3, RD150, RD152, RD181, RD311 |
| S22 | 1.1.1 | RD715, RD147c, RDbovis_wbbl2, RD239, RD11, RD131e, RD3, RD313, RD236a |
| S23 | 1.1.1 | RD715, RD147c, RDbovis_wbbl2, RD239, RD11, RD236a, RD131e |
| S24 | 2.2.1 | RD105, RD715, RD3, RD152, RD181, MiD1 |
| S25 | 2.2.1 | RD105, RD715, RD131e, RD3, RD152, RD181, RD131e, RD207 |
| S26 | 2.2.1 | RD105, RD715, RD3, RD152, RD181, RD311 |
| S27 | 2.2.1.1 | RD105, RD715, RD3, RD150, RD152, RD181, RD311 |
| S28 | 1.2.1.2.1 | RD147c, Rdbovis_wbbl2, RD715, RD239, RD210 |
| S29 | 1.2.1.2.1 | RD147c, Rdbovis_wbbl2, RD715, RD239, RD131e, RD210 |
| S30 | 1.1.1 | RD715, RD147c, RDbovis_wbbl2, RD239, RD11, RD3, RD236a, RD131e |
| S31 | 1.2.1.2.1 | RD147c, Rdbovis_wbbl2, RD715, RD239, RD131e, RD210 |
| S32 | 1.2.1.2.1 | RD147c, Rdbovis_wbbl2, RD715, RD239, RD131e, RD210 |
| S33 | 1.1.1 | RD715, RD147c, RDbovis_wbbl2, RD239, RD11, RD3, RD236a, RD131e, DS70 |
| S34 | 2.2.1 | RD105, RD715, RD152, RD3, RD311, RD181, MiD1 |
| S35 | 2.2.1 | RD105, RD715, RD152, RD3, RD311, RD181, MiD1 |
| S36 | 1.1.1 | RD715, RD147c, RDbovis_wbbl2, RD239, RD11, RD236a, RD131e |
| S37 | 2.2.1 | RD105, RD715, RD152, RD3, RD311, RD181, MiD1 |
| S38 | 1.2.1.2.1 | RD147c, Rdbovis_wbbl2, RD715, RD239, RD131e |
| S39 | 1.1.1 | RD715, RD147c, RDbovis_wbbl2, RD239, RD11, RD3, RD313, RD236a, RD131e |
| S40 | 1.2.1.2.1 | RD147c, Rdbovis_wbbl2, RD715, RD239, RD210 |
| S41 | 1.1.1 | RD715, RD147c, RDbovis_wbbl2, RD239, RD11, RD3, RD236a, RD131e, DS70 |
| S42 | 2.2.1 | RD105, RD715, RD3, RD152 |
| S43 | 1.1.1 | RD715, RD147c, RDbovis_wbbl2, RD239, RD11, RD3, RD236a, RD131e |
| S44 | 1.1.1.1 | RD715, RD147c, RDbovis_wbbl2, RD239, RD11, RD3, RD236a, RD131e |
| S46 | 1.1.1 | RD715, RD147c, RDbovis_wbbl2, RD239, RD11, RD3, RD236a, DS70, MiD4 |
| S47 | 1.2.1.2.1 | RD147c, Rdbovis_wbbl2, RD715, RD239, RD210 |
| S48 | 1.1.1 | RD715, RD147c, RDbovis_wbbl2, RD239, RD11, RD3, RD131e, DS70 |

| | | |
|---|---|---|
| S49 | 2.2.1 | RD105, RD715, RD3, RD152, RD181, RD311 |
| S50 | 2.2.1 | RD105, RD715, RD3, RD152, RD181, RD311 |
| S52 | 1.1.1 | RD715, RD147c, RDbovis_wbbl2, RD239, RD11, RD3, RD236a |
| S53 | 4.5 | RD715, RD122, RD147c, RDbovis_wbbl2, RD239 |
| S54 | 2.2.1.2 | RD105, RD715, RD301, RD142, RD3, RD152, RD181, RD311 |
| S55 | 2.2.1.1 | RD105, RD715, RD3, RD150, RD152, RD181, RD311 |
| S56 | 1.1.1 | RD715, RD147c, RDbovis_wbbl2, RD239, RD11, RD3, RD236a, DS70 |
| S58 | 2.2.1 | RD105, RD715, RD3, RD152, RD181, RD311 |
| S60 | 2.2.1 | RD105, RD715, RD181 |
| S62 | 2.2.1 | RD105, RD715, RD3, RD152, RD181, RD311 |
| S63 | 2.2.1 | RD105, RD715, RD3, RD152, RD181, RD131e |
| S64 | 2.2.1 | RD105, RD715, <u>RD152, RD181</u> |
| S65 | 1.1.1 | RD715, RD147c, RDbovis_wbbl2, RD239, RD11, RD236a |
| S66 | 1.1.1 | RD715, RD147c, RDbovis_wbbl2, RD239, RD11, RD236a, DS70 |
| S67 | 3 | RD715, RD152, RD750, RD316, N-RD25_tbA |
| S68 | 2.2.1 | RD105, RD715, RD181, RD311 |
| S69 | 2.2.1 | RD105, RD715, RD3, RD152, RD181, RD311 |
| S70 | 2.2.1 | RD105, RD715, RD3, RD152, RS311 |
| S71 | 1.2.1.2.1 | RD147c, Rdbovis_wbbl2, RD715, RD239, <u>RD131e</u> |
| S72 | 2.2.1 | RD105, RD715, RD3, RD152, RD181, <u>RD131e</u> |

*** according to RDScan; ** underlined if not confirmed by Illumina**

**Table S4**
**Deletion analysis of Structural Variants in *pe/ppe* genes**

| *Gene (locus)* | *SV identified* | *Lineage (n)* | *Known** |
|---|---|---|---|
| *pe_pgrs1 (Rv0109)* | Truncated | 1.1.1 (1/17) | |
| *pe_pgrs2 (Rv0124)* | Truncated | 1.2.1.2.1 (10/10) 1.2.1.2 (1/1) | Deleted in L6 (RD701), truncated in L4.3.3 |
| *pe_pgrs3 (Rv0278c)* | Gene fusion | 2 (25/25) | Gene fusion with *pe_pgrS4* in L2 |
| *pe_pgrs4 (Rv0279c)* | Gene fusion | 2 (25/25) | Gene fusion with *pe_pgrS3* in L2 |
| *ppe5 (Rv0304c)* | Deletions | 1.1.1 (2/17), 2.2.1 (1/20) | Truncated in L5/6/*bovis* and in sporadic samples |
| *ppe6 (Rv0305c)* | Small deletion | 2.2.1 (2/20) | Deletions in several sub-lineages |
| *ppe8 (Rv0355c)* | Truncated and Deletion | 1 (29/30), 2 (24/25), 4.2 (1/1) | Truncated in some L2, and deletion in some ancient lineages |
| *pe_pgrs6 (Rv0532)* | Truncated | 1.2.1.2.1 (10/10) | Truncated in ancient lineages |
| *pe_pgrs7 (Rv0578c)* | Deletions | Large (1.1.1 (6/17)) Small (1.2.1.2.1 10/10, 1.2.1.2 1/1) | |
| *pe_pgrs8 (Rv0742)* | Truncated | 1 (30/30) | |
| *pe_pgrs9 (Rv0746)* | Truncated | End(1 30/30) Beginning(2 25/25) | Truncated in sporadic samples |
| *pe_pgrs10 (Rv0747)* | Truncated | 1.2.1.2.1 (2/10), 2.2.1 (16/20), 2.2.1.1 (3/3), 3 (1/1) | Truncated in L5 and sporadic samples |
| *pe_pgrs13 (Rv0833)* | Truncated | 1.1.1 (2/17), 1.2.1.2.1 (2/10), 2 (17/25), 4.2 (1/1) | Gene fusion with *pe_pgrs12* in ancient lineages, truncated in some L2 and sporadic samples |
| *pe_pgrs16 (Rv0977)* | Truncated | 1.1.1 (6/17), 1.2.1.2.1 (1/10) | Truncated in L4.1 |
| *pe_pgrs19 (Rv1067c)* | Gene fusion (L1) | 1 (30/30) | Gene fusion with *pe_pgrs20* in L1 |
| *pe_pgrs20 (Rv1068c)* | Gene fusion (L1) | 1 (30/30) | Gene fusion with *pe_pgrs19* in L1 |
| *pe_pgrs21 (Rv1087)* | Missing (L3) Truncated (L2) Large Deletion | 1.1.1 (2/17), 1.2.1.2.1 (2/10), 2 (18/25), 3 (1/1), 4 (2/3) | Differences in sequence in L3 |
| *pe_pgrs22 (Rv1091)* | Truncated | 1 (30/30) | Truncated in L1.1.3 and L5 |
| *pe_pgrs23 (Rv1243c)* | Large deletion and truncated | Large ( 1.2.1.2.1 10/10 1.2.1.2 1/1) Truncations (2 25/25) | Truncated in L3 |
| *pe_pgrs24 (Rv1325c)* | Large deletion and Truncated | 1.1.1 (1/17) 1.1.3.1 (1/1) | |
| *pe_pgrs26 (Rv1441c)* | Truncated | 1.1.1 (7/17), 2.2.1 (2/20), 4 (2/3) | |
| *pe_pgrs27 (Rv1450c)* | Large deletion and truncated | 1.1.1 (1/17), 1.1.3.1 (1/1), 2.2.1 (3/20), 4.5 (1/1) | Truncated in some samples, different sequences |

| | | | |
|---|---|---|---|
| *ppe24 (Rv1753c)* | Truncated | 1.1.1 (2/17), 1.1.3.1 (1/1), 2.2.1 (1/20), 3 (1/1), 4.5 (1/1) | |
| *wag22 (Rv1759c)* | Deletion | 2 (25/25), 3 (1/1), 4 (3/3) | Deleted in some samples (RD152) |
| *ppe25 (Rv1787)* | Truncated | 1.2.1.2.1 (1/10) | Deleted in some samples |
| *pe_pgrs33 (Rv1818c)* | Truncated | 1.2.1.2.1 (10/10), 1.2.1.2 (1/1), 3 (1/1), 4 (2/3) | Truncated in L1 |
| *ppe34 (Rv1917c)* | Truncated | 1 (25/30), 2.2.1 (2/20), 2.2.1.1 (1/3), 2.2.1.2 (1/1), 3 (1/1), 4 (2/3) | Truncated in most lineages due to IS6110 |
| *pe_pgrs35 (Rv1983)* | Missing | 1.1.1.1 (1/1) | Missing in sporadic samples |
| *ppe38 (Rv2352c)* | Deletion | 1.1.3.1 (1/1), 2.2.1 (1/20) | Deletion in L2 (RD185) |
| *ppe39 (Rv2353c)* | Deletion | 1.1.3.1 (1/1), 2.2.1 (1/1) | Deletion of beginning of the gene in most isolates |
| *ppe40 (Rv2356c)* | Truncated | 1.1.3.1 (1/1), 2.1 (1/1), 2.2.1 (1/20) | Truncated in sporadic samples (IS6110) |
| *pe_pgrs45 (Rv2615c)* | Deletions | Large (2.2.1 (3/20)<br>Small (2 24/25) | |
| *pe27 (Rv2769c)* | Deleted | 2.2.1.1 (1/3) | |
| *ppe44 (Rv2770c)* | Deleted | 2.2.1.1 (1/3) | |
| *ppe46 (Rv3018c)* | Deleted | 1.1.1 (1/17), 1.1.3.1 (1/1), 1.2.1.2 (1/1), 4.8 (1/1) | Truncated in 4.1.1.3 (IS6110) and in other sporadic samples |
| *pe27a (Rv3018A)* | Deleted | 1.1.1 (1/17), 1.1.3.1 (1/1), 1.2.1.2 (1/1), 4.8 (1/1) | Deleted in some samples |
| *esxS/pe28 (Rv3020c)* | Deleted | 1.1.1 (1/17), 1.1.3.1 (1/1), 1.2.1.2 (1/1), 2.2.1 (1/20), 4 (2/3) | Deleted in some samples |
| *ppe47 (Rv3021c)* | Deleted | 1.1.1 (1/17), 1.2.1.2 (1/1) | Pseudogene |
| *ppe48 (Rv3022c)* | Deleted | 1.1.1 (1/17), 1.2.1.2 (1/1) | Pseudogene |
| *pe29 (Rv3022A)* | Deleted | 2.2.1 (2/20) | |
| *ppe50 (Rv3135)* | Deleted | 1 30/30 | Deletion in L1 |
| *ppe52 (Rv3144c)* | Truncated | 1.1.3.1 (1/1) | Truncated in some L3 |
| *ppe53 (Rv3159c)* | Truncated | 1.1.1 (17/17), 1.1.1.1 (1/1) | Truncated across most lineages |
| *ppe54 (Rv3343c)* | Truncated | 1.1.1 (1/17), 1.2.1.2.1 (1/10), 2.2.1 (2/20), 3 (1/1) | Truncated in sporadic samples (IS6110/big insertions) |
| *pe_pgrs49 (Rv3344c)* | Truncated | 1.1.1 (4/17), 1.2.1.2.1 (10/10), 2 (23/25), 3 (1/1) | Gene fusion with *pe_pgrs*50 across several lineages |
| *pe_pgrs50 (Rv3345c)* | Deletion | 1 (30/30), 2.1 (1/1), 2.2.1 (6/20) | Truncated in L1 and some L2; |

| | | | |
|---|---|---|---|
| *ppe55 (Rv3347c)* | Large deletion | 2.2.1 (1/1) | Truncated in sporadic samples |
| *ppe56 (Rv3350c)* | Truncated | 1.1.1 (5/17) | Truncated in L2 and L6 |
| *pe_pgrs52 (Rv3388)* | Truncated | 1.2.1.2 (1/1), 1.2.1.2.1 (8/10), 2.2.1 (1/30) | Truncated in sporadic samples |
| *pe_pgrs53 (Rv3507)* | Truncated | 1 (19/30), 2 (17/25), 4.2 (1/1) | Truncated in L5 and some L2 |
| *pe_pgrs54 (Rv3508)* | Truncated | 1 (26/30), 2 (16/25), 4 (2/3) | Truncated in L6, some L3, and sporadic samples |
| *pe_pgrs55 (Rv3511)* | Gene fusion | 1.1.1 (1/17), 1.2.1.2.1 (1/10) | Truncated in L5; gene fusion with *pe_pgrs56* in some other samples |
| *pe_pgrs56 (Rv3512)* | Gene fusion | 1.1.1 (1/17), 1.2.1.2.1 (1/10) | Gene fusion with *pe_pgrs55* in some samples |
| *pe_pgrs57 (Rv3514)* | Truncated | 1.1.1 (11/17), 1.2.1.2 (1/1), 1.2.1.2.1 (5/10), 2.2.1 (1/1), 4.5 (1/1) | Truncated in L6, most of L2, and in sporadic samples |
| *ppe66 (Rv3738c)* | Deletion | 2.1 (1/1)<br>3 (1/1) | Deleted in L3 |
| *ppe67 (Rv3739c)* | Truncated | 3 (1/1) | Truncated in L3 |

*** found in Gómez-González et al. [12]**

## Figure S1

## Whole genome analysis pipeline

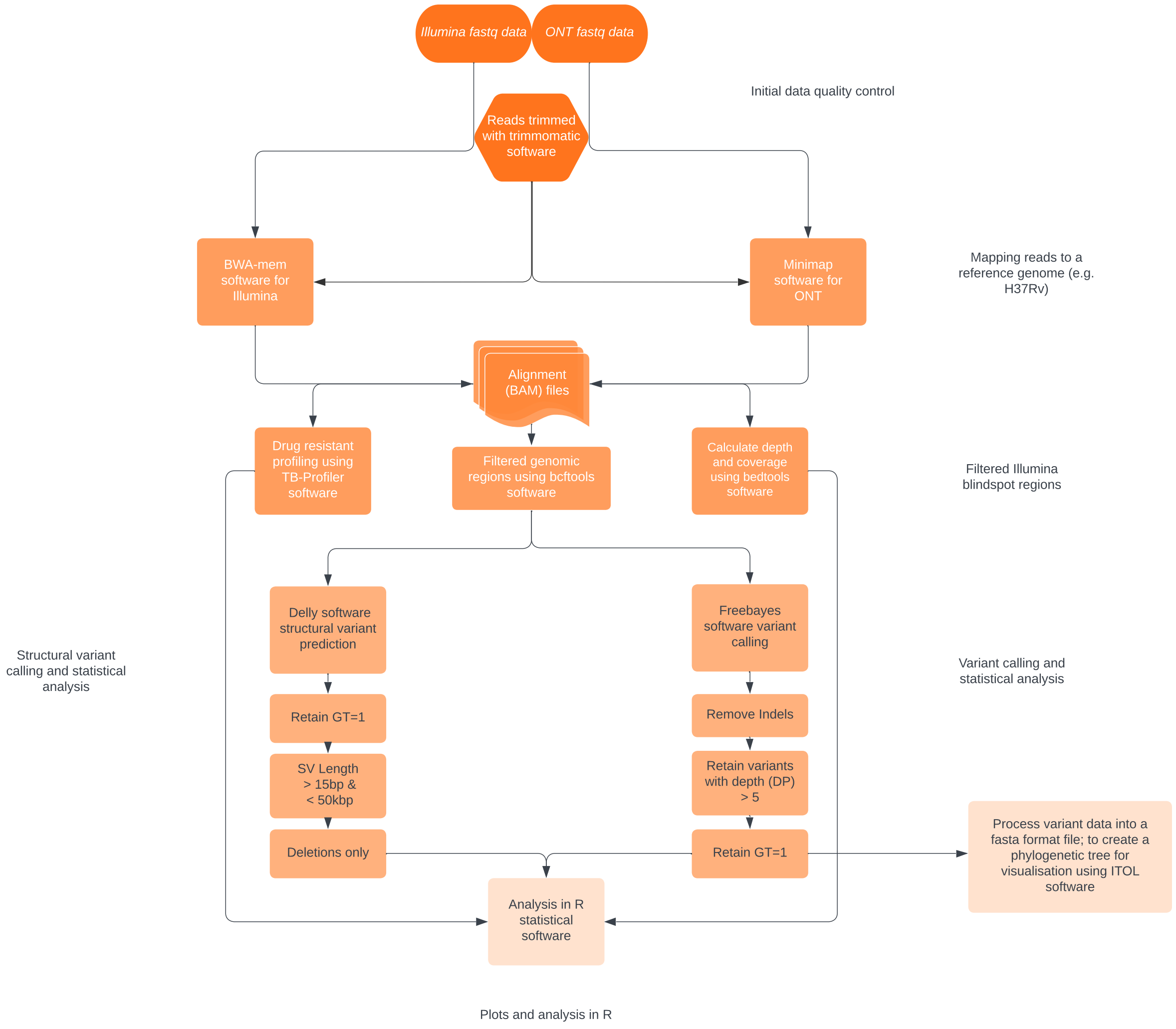


ONT = Oxford Nanopore Technology; DP = read depth parameter; GT = genotype parameter; indels = insertions and deletions; SV = structural variant

**Figure S2**

**Comparison of average percentage coverage of genes between Illumina and Oxford Nanopore Technology (ONT) platforms, coloured by drug resistance (red), *pe/ppe* (blue) or other (black) gene category.**

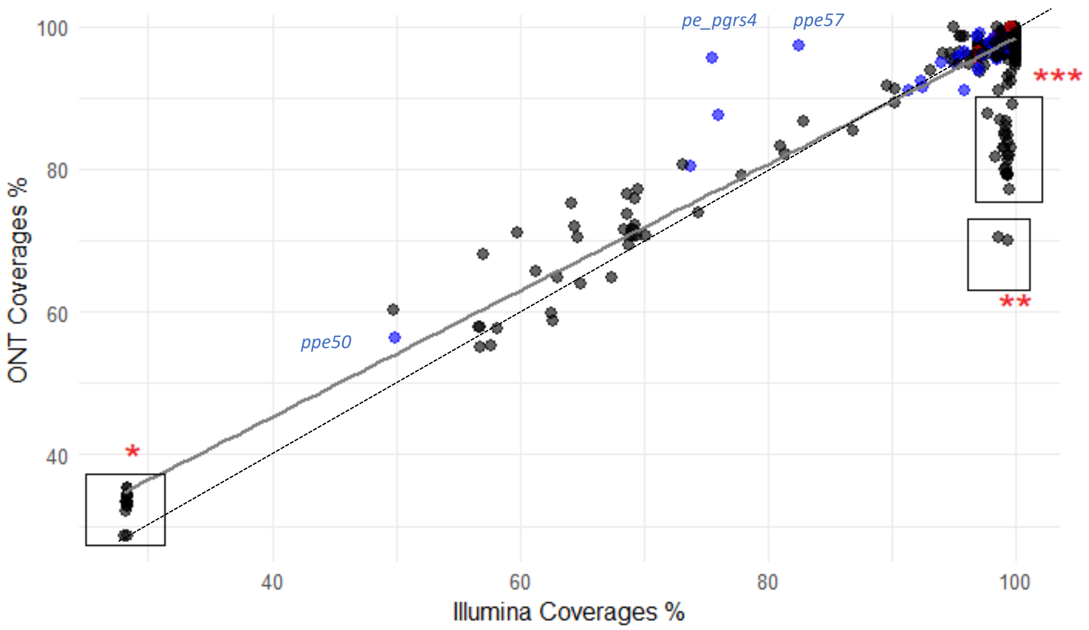


* Genes within the region *Rv1573* to *Rv1586* (includes deletion RD3 linked to lineage 2)

** Genes *Rv3474* and *Rv3475*

*** Genes *Rv0795, Rv0796, Rv1369c, Rv1370c, Rv1765, Rv2105, Rv2167, Rv2168c, Rv2479c, Rv2648, Rv2649, Rv3184, Rv3185, Rv3187, Rv3380*, and *Rv3381*

**Figure S3**

**Average coverage in genes across all 59 *M. tuberculosis* isolates for Illumina (top) and Oxford Nanopore Technology (bottom) platforms. Low coverage areas coincide with lineage-specific regions of difference (large deletions)**

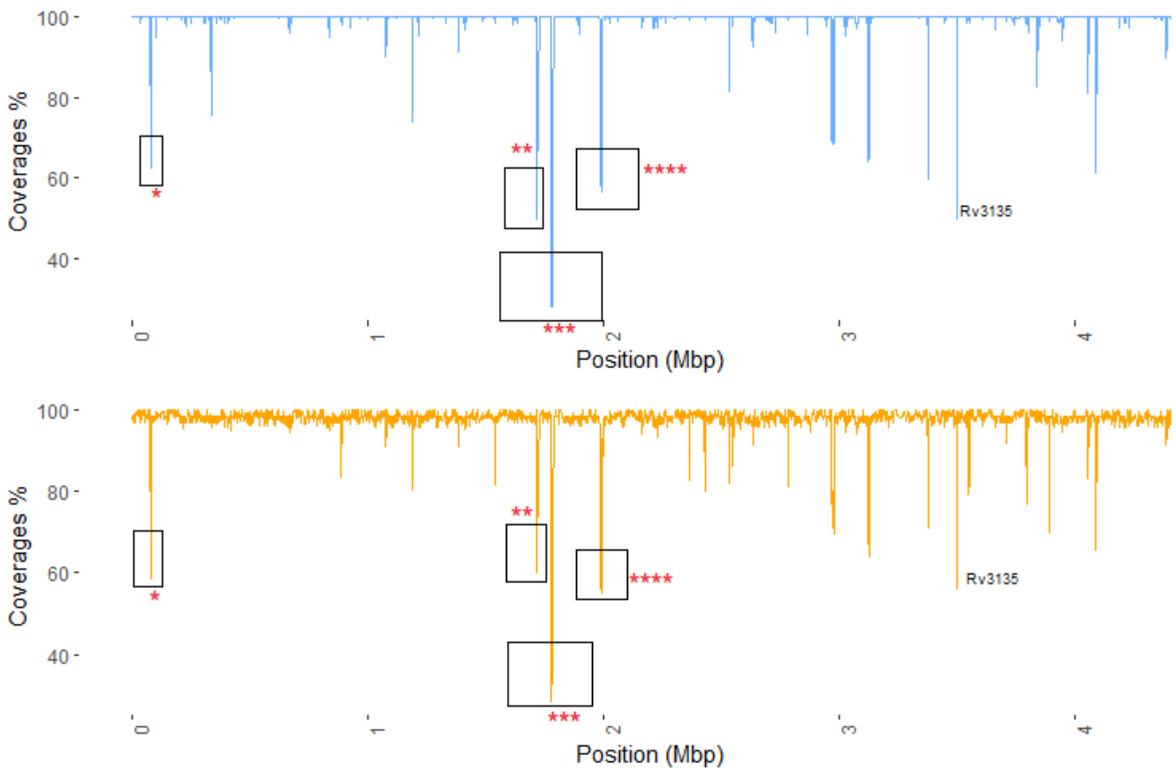


* *Rv0072* and *Rv0073*, ** *Rv1524* and *Rv1525*, *** *Rv1573-Rv1586*, **** *Rv1758-Rv1762c*
